# A rare case of Niemann–Pick disease type C without neurological involvement in a 66-year-old patient

**DOI:** 10.1016/j.ymgmr.2015.02.004

**Published:** 2015-03-06

**Authors:** C.R. Greenberg, J.G. Barnes, S. Kogan, L.E. Seargeant

**Affiliations:** a Department of Pediatrics and Child Health, University of Manitoba, Winnipeg, Canada; b Department of Neurology, University of Manitoba, Winnipeg, Canada; c Department of Ophthalmology, University of Manitoba, Winnipeg, Canada; d Diagnostic Services Manitoba, Winnipeg, Canada

**Keywords:** Niemann–Pick disease type C (NP-C), Splenomegaly, Organomegaly, Asymptomatic

## Abstract

The case of a 66 year-old female — the oldest known living patient with Niemann–Pick disease type C (NP-C) who remains free of any neurological or psychiatric manifestations 18 years after presentation — is presented. An incidental finding of massive splenomegaly was detected during a routine pelvic ultrasound. The pathology report after splenectomy showed the presence of lipid-laden macrophages. Fibroblasts cultured in LDL-enriched medium revealed abnormal filipin staining consistent with cholesterol-filled vesicles and the rate of cholesterol esterification in response to stimulation of LDL-cholesterol uptake was significantly depressed at 6% of that seen in cells from normal controls, but at a level similar to that observed in an NP-C positive control. Molecular genetic testing later revealed a compound heterozygous mutant NP-C genotype comprising two previously described disease-causing mutations in the *NPC1* gene, one in exon 8 (c.1133T>C [V378A]) and one in exon 13 (c.1990G>A [V664M]). These findings confirmed the diagnosis of NP-C. Only three patients with this disorder aged > 53 years have previously been reported, all of whom presented with neurological or neuropsychiatric manifestations. Our patient is the first reported NP-C patient, now in her seventh decade of life, who has to date only manifested splenomegaly. This case highlights the extreme clinical variability of NP-C, and the need to consider this disease in the differential diagnosis of organomegaly, even in the absence of neurological, psychiatric and related clinical signs.

**Synopsis:**

An elderly female patient with confirmed NP-C and isolated splenomegaly has remained asymptomatic for neurological, cognitive, psychiatric or ophthalmologic abnormailities into her seventh decade of life.

## 1 Introduction

Niemann–Pick disease type C (NP-C) is a rare autosomal recessive lysosomal storage disorder (LSD) caused by mutations in the *NPC1* gene (in 95% of cases) or the *NPC2* gene. The signs and symptoms and natural history of NP-C are extremely heterogeneous, which pose a major challenge to diagnosis [7]. The disease is usually characterized by progressive neurological deterioration and a variable pattern of visceral symptoms [13]. In infants and children, classic NP-C is often associated with cholestatic liver disease, isolated splenomegaly or hepatosplenomegaly, while in adult hepatosplenomegaly, if present, is usually asymptomatic and often goes unrecognized. In terms of common neurological signs, vertical supranuclear gaze palsy (VSGP), cerebellar ataxia and gelastic cataplexy are considered strongly suggestive of NP-C. However, many of the neurological and psychiatric signs of the disease (e.g., behavioral disturbances, cognitive decline and psychosis) are shared with other conditions that involve progressive neurological deterioration, and NP-C is therefore often overlooked [2], [7], [14].

The age at onset of neurological signs and symptoms is considered to exert a strong influence on disease progression and prognosis in NP-C [15]. Epidemiological studies have led to the definition of early infantile- (at < 2 years of age), late infantile- (at age 2 to < 6 years), juvenile- (at age 6 to < 15 years) and adolescent/adult-onset (≥ 15 years) forms of the disease [7], [13], [15]. The infantile- or juvenile-onset forms represent the majority of reported cases [8], but increasing numbers of adolescent and adult patients are being diagnosed [1], [10], [13].

The filipin staining test traditionally used as the standard method for diagnosing NP-C [13]. However, filipin staining is time-consuming and expensive, and more and more patients are now diagnosed by molecular genetic testing [1], [7]. Here, we describe an adult female patient with isolated splenomegaly and confirmed NP-C who has not displayed any neurological, cognitive or ophthalmologic abnormalities during 18 years of follow-up. Such patients have not been reported previously in the literature and, as such, are not included in current clinical management guidelines for NP-C [7].

## 2 Case presentation

A Caucasian woman of non-Ashkenazi Jewish ancestry underwent a splenectomy at 48 years of age due to massive splenomegaly. The splenomegaly was an incidental finding during a routine pelvic ultrasound. Her past medical history was unremarkable except for hypercholesterolemia and type 2 diabetes (both of which were medically controlled), and bilateral knee replacements. Her extended family history did not indicate any contributory factors, and her parents were of European descent and not known to be consanguineous. Two male siblings were unaffected, as were two adult daughters.

At the time of the patient's splenectomy, the spleen weighted was grossly enlarged with a weight of 788 g, (normal — 150 to 200 g for healthy adults). Other than the increased size and lipid-laden macrophages, histology was unremarkable. No further investigations were pursued until the patient was referred to the adult metabolic clinic 9 years after splenectomy regarding suspected Gaucher disease type 1. Physical examination at the time of her initial evaluation revealed that she was moderately obese, with a body weight of 91.6 kg. Neurologic and ophthalmologic examinations were unremarkable. Brain MRI was normal.

Laboratory investigations revealed a normal complete blood count with white blood cell (WBC) count being 10.6 × 10^9^/L (normal 4.5–11) and normal WBC glucocerebrosidase activity 0.28 U/g/protein (normal 0.14–0.29). Serum chitotriosidase level was also normal 44 nmol/h/mL (normal 0–150). Additional investigations for other lysosomal storage diseases were thus pursued. Fibroblasts cultured in LDL-enriched medium revealed abnormal filipin staining (Mayo Clinic Laboratories) consistent with cholesterol-filled vesicles and the rate of cholesterol esterification in response to stimulation of LDL-cholesterol uptake was significantly depressed at 6% of that seen in cells from normal controls, but at a level similar to that observed in an NP-C positive control. Molecular genetic testing revealed a compound heterozygous mutant NP-C genotype comprising two previously described mutations in the *NPC1* gene, one in exon 8 (c.1133T>C [V378A]) and one in exon 13 (c.1990G>A [V664M]), establishing the diagnosis of NPC [1], [5]. Although both mutations are described as mild there are known to be disease-causing. The exon 8 c.1133T>C (V378A) mutation is found in Loop C (lumenal domain 2) and human disease-causing mutations in NPC1 luminal domain 2 decrease NPC2 binding and support the model in which the NPC1 domain 2 holds NPC2 in position to facilitate directional cholesterol transfer from NPC2 onto NPC1 protein for export from lysosomes [6]. The second mutation, in exon 13 c.1990G>A (V664M) is found in transmembrane domain (TM) 4 and is on the list of known NPC1 disease-causing point mutations [11].

The patient was followed annually with no symptoms or signs of disease progression. Patient lived independently and was employed fulltime as a companion for disabled people. Recently (2015) formal neurological and ophthalmological reassessments were performed. There was normal cognitive function with a score of 30/30 on the Mini Mental Status Examination (MMSE). Beck depression inventory (BDI) score did not suggest any mood disorder. Cranial nerves were normal including a dilated fundoscopic examination and there is no limitation of upward or downward gaze, optokinetic responses were normal and the vestibular-ocular reflex was preserved. Muscle bulk, tone, and strength were normal as were her gait and coordination with a negative Romberg's test. Deep tendon reflexes were present and equal, plantar responses were downgoing and clonus was not elicited. Sensory examination was normal including normal vibration and position senses. Caloric testing was declined by the patient.

Oxysterol assays on two separate blood samples from this patient around this time revealed normal cholestanetriol (oxysterol) levels of 0.042 and 0.040 ng/μL (normal < 0.05) [3], [9].

Now for a total of 18 years of follow-up post-splenectomy, the patient has remained asymptomatic with no abnormal neurological, neuropsychiatric, or ophthalmological signs. There has also been no progression of visceral symptoms of NP-C, and she has not received any oral substrate reduction therapy [4], [7] to date.

## 3 Discussion

This patient does not demonstrate the classic clinical phenotype of progressive multisystem disease that normally characterizes NP-C. This case therefore emphasizes the broad spectrum of NP-C phenotypes, which is known to include patients with asymptomatic splenomegaly. It also highlights gaps in our knowledge regarding the natural history of the disease.

Although comparable in some respects to previously published patients with visceromegaly, particularly those with adolescent/adult-onset disease, psychiatric and or neurological manifestations typically develop at some point [10], [12], [13]. So far as we are aware the current case is the only NP-C patient reported who has manifested only isolated splenomegaly up to such an advanced age; she is now into her seventh decade of life. In addition, while recent evidence suggests that plasma oxysterols may serve as a valuable marker for screening and, possibly, monitoring NP-C in the majority of patients ([3]; Porter et al., 2010), this case suggests that oxysterol analysis as a screening tool might not have 100% sensitivity, or may only detect abnormalities in patients with neurological involvement.

NP-C needs to be considered in the differential diagnosis of isolated splenomegaly in adults without neurological and neuropsychiatric manifestations and, in the absence of elevated oxysterols, molecular analysis of *NPC1* and *NPC2* genes should be pursued.

## Conflict of interest

Cheryl Greenberg has received research funding (NIH Clinical Trials NCT01176266 and NCT01163149), speaker honoraria from Alexion Pharmaceuticals Ltd. Jeffrey Barnes, Sylvia Kogan and Lorne Seargeant have no conflicts to declare.

## Informed consent

All procedures followed were in accordance with ethical standards of the institutional committee and best clinical practice, and with the Helsinki Declaration of 1975, as revised in 2000. Informed consent was obtained from the patient for publication of this report.

## Contributions of individual authors

There are no known previous or simultaneous publications similar to this case. The submitting author secured the approval of the final draft from all co-authors before article submission. All co-authors have been involved with the analysis and interpretation of these clinical data, have provided important critical review/input during preparation of the manuscript, and agreed to submission. Informed consent was obtained from the patient before submission of this case report for publication.
